# Enclosure Background Preferences Differ between Sexes and Color Morphs in the Gouldian Finch

**DOI:** 10.3390/ani13081353

**Published:** 2023-04-15

**Authors:** Robert I. Moise, Georgina R. Eccles, Claudia Mettke-Hofmann

**Affiliations:** 1 School of Biological and Environmental Sciences, Liverpool John Moores University, Liverpool L3 3AF, UK; 2 Caesar Kleberg Wildlife Research Institute, Texas A&M University-Kingsville, Kingsville, TX 78363, USA

**Keywords:** background matching, camouflage, animal welfare, exploration, neophilia, color polymorphism

## Abstract

**Simple Summary:**

Most animals blend well into their natural environment, which protects them from predators. However, in captive environments, animals are often exposed, which can lead to stress. Research suggests that animals are more difficult to detect in complex environments. We tested background preferences in the Gouldian finch, which occurs in two main head color morphs in the wild. Birds were tested in groups of four in either the same head color (black or red) or mixed head color (two black and two red) pairings. One half of the cage had a simple background pattern, the other a complex background pattern. We measured the time spent in front of each background after 10 days in the cage (phase 1), after which backgrounds were swapped (left to right), and assessed preferences again on day 17 (phase 2). Birds preferred the simple background, particularly in phase 1. However, females initially chose the simple background but used both backgrounds in phase 2, whereas changes in males were not significant. Both color morphs preferred the simple background in phase 1, with the black-headed birds moving to the complex background in phase 2. Results indicate that background preferences differ among individuals, which should be considered when designing enclosures.

**Abstract:**

Most wild animals camouflage well into their environment, providing protection from predators, whereas captive animals often contrast with their background. This can cause stress for the animal, which may perceive it as being exposed. Theory suggests that prey is more difficult to detect in front of complex backgrounds; hence, animals should prefer complex over simple backgrounds. We tested this in the polymorphic Gouldian finch by providing a complex background pattern in one half of the flight cage and a simple background pattern in the other half for 10 days (phase 1). Patterns were then swapped and presented for another week (phase 2). Groups of four birds consisting of either pure black-headed or red-headed or mixed head color (two black-headed and two red-headed) pairings were tested. Gouldian finches spent significantly more time in front of the simple background in phase 1 but not in phase 2. Specifically, females preferred the simple background in phase 1 significantly more than males. Moreover, red-headed birds consistently perched in front of the simple background, whereas black-headed birds used both backgrounds, particularly in phase 2. Results indicate that background preferences differ between sexes and morphs, which should be considered when designing backgrounds. Moreover, natural habitat preferences need consideration.

## 1. Introduction

One of the main anti-predator strategies is camouflage, with animals blending into their environment. As most predators use vision to detect prey with a preference for color cues [1,2] (but see [3]), animals’ coloration has evolved to match their background [4,5,6,7,8]. However, in captive environments, there is often a mismatch between an animal’s color and its environment, which can be perceived by the animal as being vulnerable and under stress.

Background matching is one of the most common camouflage strategies [8,9,10,11,12,13,14]. Camouflage is achieved by matching the average background [11,15,16] or specific aspects of the background (e.g., leaves) [17] and exploiting the perception and cognitive mechanisms of predators [18]. Reducing the contrast against the background has been shown to decrease detectability and hence predation [6,8,19,20,21]. It can affect population densities with higher densities in areas of better camouflage efficiency [16], support species divergence with recently diverged species showing a close match to the environment [20], and positively affect the invasion success of new habitats with better background matching. Moreover, it can prolong foraging [22], reduce social harassment, or facilitate hidden copulations [23]. While it is usually prey that camouflages to avoid predation, predators also camouflage to get close to prey [24] or avoid mobbing [19].

Considerable attention has been paid to color polymorphic species, where individuals in a population differ in their appearance, which can affect their camouflage [25,26]. For example, in the color polymorphic cichlid *Amphilophus labiatus*, morphs differed in their ability to match backgrounds, which coincided with their frequency [27]. Likewise, in Tawny owls (*Strix aluco*), the gray morph was less conspicuous against a snowy background and tree trunks, resulting in higher survival in snowy winters [19,21]. In other species, morphs match different backgrounds, affecting their microhabitat selection [28,29].

In captivity, animals often contrast against their background. The inability to camouflage with the background has been shown to cause stress and negative thigmotaxis in European cuttlefish (*Sepia officinalis*) [30] and stress, atypical behavior, and weight loss in African clawed frogs (*Xenopus laevis*) [31]. When given the choice, hermit crabs (*Pagurus bernhardus*) prefer shells that match the background [32], desert tortoises (*Gopherus agassizii*) preferentially rest next to rocks where they are more difficult to detect [10], and Gouldian finches (*Chloebia gouldiae*) prefer green over white backgrounds, which matches their color better [33]. Both 2D [30,33] and 3D backgrounds (e.g., [31]) are effective. This indicates that welfare is improved in captive animals when they can blend in with the background [31]. However, finding the right background may be difficult given that individuals may differ in their coloration (males vs. females, young vs. adults, color polymorphic species). One solution may be to increase the complexity of the enclosure.

Complex environments increase the background noise against which predators must detect prey [18]. While background matching is still important [4,7,23], complexity has been shown to reduce predation [4,34,35,36,37,38,39]. Moreover, Dimitrova and Merilaita [40] found that complex backgrounds reduce predation for matching and non-matching prey, whereas Maend et al. [41] demonstrated that complex backgrounds eliminate size-dependent predation. In a captive environment, Jacky dragons (*Amphibolurus muricatus*) preferred perches with more complex patterns over simply patterned ones [14]. In contrast, Gouldian finches preferentially perched in front of simple green backgrounds and avoided complex patterned backgrounds, possibly due to their open habitat preference [33].

Here we re-investigate preferences for simple and complex backgrounds in the Gouldian finch by controlling for the overall appearance of the backgrounds to account for their open habitat preference. Gouldian finches are color polymorphic in both sexes, with a ratio of about 70% black-headed birds, 30% red-headed birds, and less than 1% yellow-headed birds in the same population [42]. They inhabit the open savannah woodland of Northern Australia [43] and are listed as endangered by the Australian Government, with just 2500 birds left in the wild due to habitat destruction [44]. Gouldian finches are one of the most popular bird species among aviculturists [45], and self-sustaining populations have been maintained in captivity since the Australian export ban in 1960 [46]. Research has shown that the Gouldian finch’s head color indicates their personality. Red-headed birds are more aggressive [47,48,49] but take less risk in potentially dangerous situations and are less explorative of changes in their environment than black-headed birds [49,50]. However, red-headed birds enter unfamiliar environments faster and accept novel food quicker than black-headed birds [51,52]. Earlier experiments on background matching have shown that Gouldian finches prefer a simple green background over a patterned multi-color background [33]. However, it was unclear whether this was potentially affected by the denser appearance of the patterned background. As the Gouldian finch is an open habitat specialist [53,54], they may have chosen the appearance of openness over complexity.

In the current study, we controlled for density effects and offered two types of background—a complex and a simple—to investigate (a) whether Gouldian finches prefer complex backgrounds when density effects are controlled for, (b) how preferences may change over time, and (c) how head color, age, and sex affect preferences.

## 2. Materials and Methods

Twenty-four Gouldian finches were part of this study, consisting of 12 females (6 black-headed and 6 red-headed) and 12 males (6 black-headed and 6 red-headed), ranging in age from one to seven years. All birds originated from 11 bird breeders purchased over the years. Birds were housed in groups of six birds mixed in age, sex, and head color in flight cages (120 cm × 80 cm × 100 cm; L × W× H). The cages contained three wooden walls plus wire mesh on the front and ceiling. The cages were furnished with natural twigs and perches. Food and water were attached to the front, with a bath on the floor. The food was a seed mixture of Blattner Amadine Zucht Spezial, Blattner Astrilden Spezial, and Blattner Rote Mannahirse (Blattner Heimtierfutter, Ermengerst, Germany). Blattner bird grit and eggshells were provided in separate pots. Birds were kept at a temperature of 24 °C and 51% humidity and provided with a full spectrum light source with a light:dark cycle of 13:11 h.

### 2.1. Experimental Procedure

**Figure 1:**
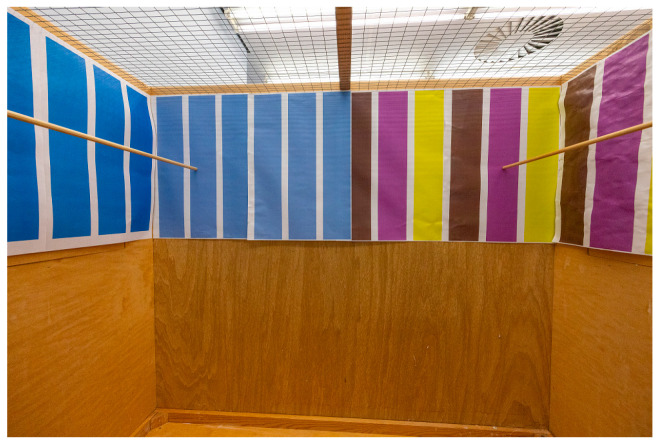
Experimental cage with different background patterns. Simple background (**left**) and complex background (**right**) are shown.

Experiments took place in an experimental room adjacent to the housing room, which contained six similarly sized flight cages (120 cm × 70 cm × 100 cm) with three wooden walls and a front and wire mesh ceiling. Cages were arranged so that birds could hear but not see each other and contained two upper perches, left and right, in the cage and a lower perch close to the bottom, which was only used by the birds when going for a bath. Food and water were provided in the middle of the wire mesh front. Each cage had two background patterns. Half of the rear wall and the adjacent side wall were covered with a simple background pattern, and the other half of the rear wall and adjacent side wall were covered with a complex background pattern (Figure 1). Backgrounds were designed in Adobe Photoshop 2018. Both background patterns consisted of vertical stripes alternating between broad (7.9 cm) and narrow (1.9 cm) stripes to control density [34]. The colors were specifically chosen to avoid green, red, and black as the backs of the birds were mostly green and the latter two differed between the color morphs. This allowed specifically testing for simple vs. complex patterns, excluding color matching, which is difficult in this colorful species. As the finches have tetrachromatic vision, including in the ultraviolet range [48], matching background colors to their plumage is further hampered. The simple background alternated broad light blue (#7d8beb) stripes with white stripes, whereas the complex background had a pattern of broad yellow (#e2eb0c), purple (#cd4fff), and brown (#5d4028) stripes separated by narrow white stripes. The lightness of backgrounds was compared using the Lab Color mode in Adobe Photoshop. The simple background had a lightness of 60 for each stripe, whereas the complex background had an overall lightness of 60 calculated as the average of yellow, brown, and purple lightness (90 + 30 + 60/3). The backgrounds were printed on 60 × 40 cm paper and attached to the walls. The position of the complex and simple backgrounds (on the left or right side of the cage) alternated between cages. A video camera was mounted in front of each cage. Overall, four cages were used at a time.

The experiments consisted of two phases: phase 1 lasted from day 1 to day 10, after which backgrounds were swapped (left-right) in each cage, and phase 2 lasted from day 10 to day 17. This allowed for controlling of side preferences. Birds were tested in groups of four same-sex individuals of different ages and head color combinations. For each sex, we had one group each of pure black-headed, pure red-headed, and mixed head colors (two birds of each head color). Groups were composed of birds, ideally from different holding aviaries, to mix birds from different groups. As age did not have an effect in the earlier study on background preferences [33], we did not control it. We tested 16 birds (distributed across four cages) in one go (batch one), followed by the remaining 8 birds (batch 2).

Birds were released into the experimental cage and allowed to habituate to the new cage environment for nine days. On day 10, birds were video recorded (GeoVision 1480, GeoVision Inc., Taipei, Taiwan) for one hour starting at 9:00 a.m. (end of phase 1). Backgrounds were swapped at 1:00 p.m. the same day, and birds were released back into the cage at 2:00 p.m., with another hour of video recording commencing at 2:00 p.m. (the start of phase 2). A third one-hour recording was performed on day 17 starting at 9:00 a.m. (the end of phase 2), after which the birds were moved back into their housing aviaries. The cages were then cleaned and prepared for the second batch of birds, which were moved in at 2:00 p.m. the same day.

### 2.2. Analysis

All analyses were performed with SPSS version 26. All data are available in the supplementary Table S1. From the videos, we extracted the total amount of time each individual spent on the perch in front of each background on day 10 (end of phase 1), again on day 10 after swapping the backgrounds (start of phase 2), and on day 17 (end of phase 2). Then the proportion of time spent in front of the simple background in relation to the overall time spent on the two perches was calculated for each of the three recording days using the formula (t(simple) − t(complex)/Σ[t(simple) + t(complex)]). Proportions were arcsine transformed to meet requirements or ANOVAs. Out of the 24 birds, two were excluded from analysis, as one had died after the first day due to unknown reasons and the other one perched primarily on the feeder.

In the first step, we evaluated potential side preferences by inspecting the proportion of time spent in front of the simple background at the end of phase 1 (day 10) and directly after the swap at the start of phase 2 (day 10). Out of the six groups tested, all the birds in one group remained on the same side of the testing cage as before (i.e., showing side preferences). This group (consisting of three birds; see above) was excluded from all further analyses.

In the second step, background preferences were analyzed (*n* = 19). The time spent in front of the simple and complex backgrounds was compared on the last day of phase 1 (day 10 before the swap) and the last day of phase 2 (day 17). Here, the actual time spent on each perch was compared rather than the proportions. As the data in phase 2 were not normally distributed, Wilcoxon tests were used.

Finally, it was investigated whether preferences changed over time (habituation) and whether this was affected by head color, age, or sex. Arcsine transformed proportions were used for this analysis comparing the proportion of time spent in front of the simple background on the last day of phase 1 (day 10) and the last day of phase 2 (day 17) using repeated measures ANOVA. The within-individual factor was phase (days 10 and 17), whereas head color, sex, and age class (4 classes: 1 year: *n* = 5, 2 years: *n* = 6, 3–4 years: *n* = 6, >4 years: *n* = 5) were the between-individual factors, which were included as main factors. Age classes were categorized in this way to have meaningful sample sizes. Two-way interactions between the within-individual factor and the between-individual factors are routinely included in the repeated measures ANOVA. Greenhouse-Geiger adjustments were used to account for deviations in the covariance matrix. Based on the outcome, we additionally tested for changes in background preferences between days 10 and 17 within sexes using paired *t*-tests, which could not be performed as part of the repeated measures ANOVA.

### 2.3. Ethics

The experiments have been in accordance with The Association for the Study of Animal Behavior (ASAB) ethical guidelines [55] and non-invasive in nature. Experiments have been approved by the University Ethics Committee (CMH_RM/2018-12).

## 3. Results

**Figure 2:**
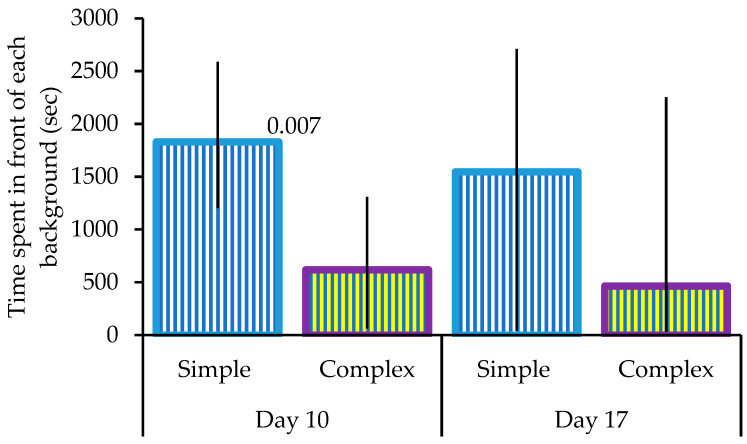
Background preferences. The median and quartiles of time spent in front of the simple and complex background at the end of phase 1 (day 10) and the end of phase 2 (day 17). Significant differences are shown.

Gouldian finches significantly preferred the simple background over the complex background at the end of phase 1 (day 10; Wilcoxon Signed Rank test: *n* = 19, *U* = −2.696, *p* = 0.007; Figure 2). This preference had disappeared by the end of the second phase (day 17; *U* = −0.845, *p* = 0.398; Figure 2), primarily due to increasing variance in the data.

**Figure 3:**
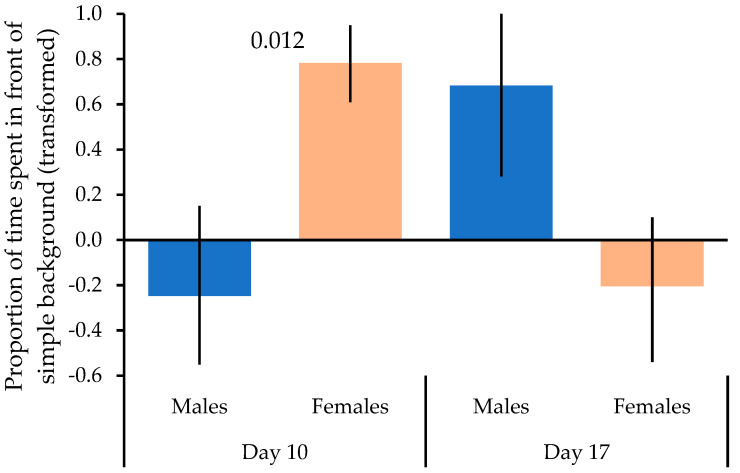
Changes in preference for a particular background over time (habituation) in relation to sex. The means ± SE of the proportions of times spent in front of the simple background are shown for males and females at the end of phase 1 (day 10) and the end of phase 2 (day 17). Significant differences from the repeated measures ANOVA are shown.

The repeated measures ANOVA investigating changes in preference over time and the effects of head color, sex, and age showed that the interaction between habituation x and sex was significant (repeated measures ANOVA: *n* = 19, *F* = 7.292, *p* = 0.018). Females spent significantly more time in front of the simple background at the end of phase 1 (day 10) as compared to the males (*t* = −2.906, *p* = 0.012), whereas the sexes did not differ significantly in their background preference from each other at the end of phase 2 (day 17; *F* = 1.531, *p* = 0.150; Figure 3). However, the paired *t*-test comparing preferences on days 10 and 17 for each sex separately indicated that females showed a significant change in preference from spending much more time in front of the simple background in phase 1 to spending similar amounts in front of both backgrounds or even a bit more time in front of the complex background in phase 2 (paired *t*-test: *t_12,1_* = 2.734, *p* = 0.019; Figure 3). In contrast, changes in preferences among males were not significant (*t_7,1_* = −1.553, *p* = 0.171; Figure 3).

**Figure 4:**
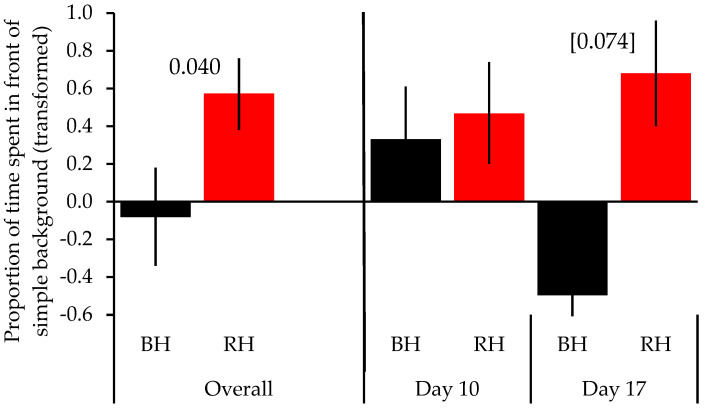
Effect of head color on background preference. The means ± SE of the proportions of times spent in front of the simple background are shown for black-headed and red-headed birds across both phases and for phase 1 (day 10) and phase 2 (day 17), separately. Significant differences are shown (square brackets indicate a strong trend).

Moreover, head color had a significant effect (*F* = 5.187, *p* = 0.040), with black-headed birds overall showing no clear preference for either background, whereas red-headed birds preferred the simple background. These differences were primarily driven by a trend among the black-headed birds to spend more time in front of the complex background at the end of phase 2 (day 17; *t* = −1.944, *p* = 0.074; Figure 4) as compared to the red-headed birds. No significant differences between head colors were present at the end of phase 1 (day 10; *t* = −0.730, *p* = 0.478; Figure 4). No other factors had an effect (Table 1).

## 4. Discussion

Overall, Gouldian finches preferred the simple over the complex background, although considerable dynamics were observed among sexes and head colors. Sexes initially differed in their background preference, with males using both backgrounds or slightly preferring the complex one, whereas females clearly preferred the simple background in phase 1. In contrast, the sexes did not differ significantly in phase 2, although females significantly changed their preferences using both backgrounds in phase 2. Likewise, red-headed birds preferred the simple background throughout the study, whereas black-headed birds used both backgrounds, particularly in phase 2.

The study specifically tested whether Gouldian finches prefer complex over simple backgrounds. While background matching with respect to colors is an important aspect of camouflage [6,8,19,20,21], we did not try to match background colors with plumage colors due to the potentially different perception of colors by the birds (tetra-chromatic vision [48]). However, the complexity of backgrounds alone has been shown to reduce predation [40]. Nonetheless, the colors might have affected background preferences. The colors in the complex pattern (yellow, violet) resembled plumage colors (belly and breast) more than the blue color of the simple pattern. One might have expected that this would further increase the preference for the complex pattern (better camouflage), which was not the case. We did not measure whether the backgrounds reflected any ultraviolet (UV). If so, birds were expected to settle in front of the background, emitting less UV, as the plumage of Gouldian finches does not reflect UV except for the turquoise band in the neck [48]. While we cannot comment on the potential UV reflectance of the backgrounds, it is unlikely that there was an effect as in both studies (Perkovic and Mettke-Hofmann [33] and this study), the Gouldian finches preferred the simple background despite the very different colors used. It is difficult to imagine that, by chance, the complex background had more UV reflectance in both studies. Finally, any differences in preferences between sexes or morphs are unlikely to be caused by the UV reflectance of the backgrounds, as sexes and morphs do not differ consistently in their UV reflectance [48]. We, therefore, believe that the differences in background preference are primarily due to the complexity of the background patterns. However, future studies should consider UV and possibly different degrees of color matching.

Gouldian finches showed a clear preference for the simple background, although this preference became non-significant in phase 2 due to increased variation. This parallels earlier findings, where background density was not controlled for [33]. However, in this earlier experiment, background preferences did not change, suggesting that the density effect of the background is one factor affecting background choice. Nonetheless, controlling for density still resulted in an overall preference for the simple background. Only a few other studies have investigated responses to complex backgrounds, with Jacky dragons preferring complexly patterned perches [14] and least killifish (*Heterandria formosa*) females choosing complex backgrounds under predation [36]. Gouldian finches live in relatively simply structured open savannah woodland [43], and the simple background seems to capture this better than the complex background. The preference for simple backgrounds aligns with the spatial exploration patterns of the Gouldian finch. They made more approach attempts (reflecting a stronger approach-avoidance conflict) in dense habitats as compared to simple novel environments before entering these habitats, supporting their open habitat preference [51]. This highlights the importance of considering the natural habitat of a species when choosing backgrounds. However, not all individuals consistently preferred the simple background.

The sexes differed in their preferences, which were modulated by different phases. In phase 1, males used both backgrounds equally, with a slight preference for the complex background, whereas females showed a clear preference for the simple background. Males are more colorful than females in the Gouldian finch and are likely to be more exposed to predation due to their higher conspicuousness [25,56,57,58]. Choosing a more complex background makes it more difficult for predators to detect them [40,41]. Males might have perceived the complex background as safer, increasing their welfare. The perception of requiring more cover may be particularly strong when new to an environment, as better camouflaged prey is less predated in new environments [20,59]. An indication for this might be that males spent more time in front of the complex background in phase 1 than in phase 2. However, this change was not significant and requires further investigation, giving the birds potentially more time to adapt. One would expect that once fully habituated to their environment, their natural preference for simple habitats would overcome their desire for higher protection. The perception of dangerousness also affected background preferences in killifish. Both males and females preferred matching over non-matching backgrounds, but only when under predation threat [36]. Moreover, females preferred complex over matching backgrounds in more dangerous situations [36].

Alternatively, males are more explorative than females in the Gouldian finch [49] and may have explored both backgrounds initially before settling on their preferred simple background. However, the first preference test was performed after 10 days, and one would expect that any exploration of a new environment would have ceased by that time. Interestingly, females started to spend more time in front of the complex background at the end of phase 2. The less conspicuous females may have felt less exposed in the novel environment and settled for their simple natural habitat preference in phase 1. Indeed, females are known to take more risk at waterholes, which was linked to their lower conspicuousness attracting fewer predators [60]. They might have relied on their lower conspicuousness in the current experiment, too. However, over time, they seemed to have explored the complex background, resulting in a significant change in preference with equal usage and even a slight preference for the complex background in phase 2. The contrasting background preferences between males and females indicate different camouflage requirements for males and females, which should be considered in enclosure design. It might also point towards different microhabitat uses in the wild [28,29]. For example, different sexes may prefer different parts of a tree. This would be an interesting aspect to investigate in the wild. Different camouflage requirements have also been shown in other species [36,61].

Finally, head color morphs differed in their background preference, with black-headed birds overall showing no preference for either background, whereas red-headed birds clearly preferred the simple background throughout the entire experiment. This is surprising as red-headed birds are more conspicuous [62], and the complex background would provide more camouflage for them [7,23], particularly as the main predators of Gouldian finches are raptors [62]. However, it is consistent with earlier findings in this species, where red-headed birds consistently preferred the simple background [33]. Again, their simple habitat preference seems to override camouflage advantages. Looking at the black-headed birds more closely, they initially also preferred the simple background but changed their preference for the complex background in phase 2. Black-headed birds are more interested in exploring changes in their familiar environment than red-headed birds [49,50]. The swap in backgrounds from left to right may have motivated the black-headed birds more to explore the complex background than the red-headed birds, which resulted in a trend for opposing background preferences at the end. Differences in microhabitat selection that aid their camouflage are well known in color polymorphic species [19,21,28,29] and should be considered when using backgrounds.

It is surprising how long the birds needed to explore the complex background, which raises the question of whether this reflects the time to habituate to the environment or their dislike of the complex background. On the one hand, the males in this experiment did not avoid the complex background in phase one, indicating that the complex background is not generally disliked. On the other hand, when this early preference for the complex background reflects the need to camouflage better in an unfamiliar environment, this supports the lengthy habituation. This would be in line with the late exploration of the complex background of the black-headed birds and the females. However, studies on vigilance show habituation to a similar novel environment within four days [63,64], which would speak against such a lengthy habituation. In the current experiment, the only difference in the experimental cages were the added backgrounds. It seems that this 2D change in the environment constituted a major change for the animals, resulting in prolonged habituation. This indicates the importance of backgrounds but also shows that backgrounds must be carefully considered. More research is needed in this area.

## 5. Conclusions

Gouldian finches preferred simple backgrounds; however, preferences differed between sexes and head color morphs, with in part opposing trends over time. Changes in preferences were likely linked to risk perception (higher in novel environments in males) and exploration later in the trial (black-headed birds and females). Considering diverting background preferences is an important aspect when designing enclosures. Specifically, a mix of backgrounds could be used to account for different camouflage requirements. Moreover, it would allow birds to choose a background reflecting their current risk perception, thereby reducing fear and increasing wellbeing. However, the results also show that natural habitat preferences require consideration, as simple, open habitat preferences seemed to have overridden the desire to camouflage better. In summary, 2D backgrounds can help improve welfare by providing choice when a mix of backgrounds is used, which addresses changing or differing camouflage requirements. However, some consideration of habitat preferences is needed.

## Figures and Tables

**Table 1:** Repeated measures ANOVA analyzed changes in background preference over time (habituation), comparing the proportion of time spent in front of the simple background at the end of phase 1 (day 10) and the end of phase 2 (day 17) in relation to head color, sex, and age class.

Multivariate Tests				
Effects	F Value	Hypothesis df	Error df	Significance
Phase	0.005	1	13	0.946
Phase × sex	7.292	1	13	0.018
Phase × head colour	0.871	1	13	0.368
Phase × age class	1.476	3	13	0.267
Tests of between-subject effects				
Intercept	3.945	1		0.069
Sex	0.564	1		0.466
Head colour	5.187	1		0.040
Age class	1.610	3		0.235

## Data Availability

All data are available as a supplement (Table S1) to this paper.

## Supplementary Materials

The following supporting information can be downloaded at: https://www.mdpi.com/article/10.3390/ani13081353/s1, Table S1: data.
